# An *in silico* approach towards identification of novel drug targets in pathogenic species of *Leptospira*

**DOI:** 10.1371/journal.pone.0221446

**Published:** 2019-08-20

**Authors:** Reena Gupta, Rashi Verma, Dibyabhaba Pradhan, Arun Kumar Jain, Amineni Umamaheswari, Chandra Shekhar Rai

**Affiliations:** 1 University School of Information, Communication & Technology, Guru Gobind Singh Indraprastha University, New Delhi, India; 2 Biomedical Informatics Centre, National Institute of Pathology-Indian Council of Medical Research, New Delhi, India; 3 Computational Genomics Centre, Indian Council of Medical Research, Campus—All India Institute of Medical Sciences, New Delhi, India; 4 Department of Bioinformatics, Sri Venkateswara Institute of Medical Sciences, Tirupati, Andhra Pradesh, India; Rajendra Memorial Research Institute of Medical Sciences, INDIA

## Abstract

Leptospirosis is one of the leading zoonotic infections worldwide. As with other infectious diseases, report of antimicrobial resistance to existing therapeutic arsenal poses challenges in the management of disease. Hence, identification of novel drug targets for the pathogen deems essential. Present study used combined approach of comparative and subtractive genomics to identify putative drug targets. Crucial genes of 16 pathogenic *Leptospira* strains were filtered and subjected to homology search *via* target identification tool “TiD”. Thereafter, comparative analysis was performed for non-homologous, essential genes to accomplish the broad-spectrum drug target. Consequently, 37 essential genes were found to be conserved in at least 10 strains of *Leptospira*. Further, prioritization of resultant set of genes revealed 18 were hubs in protein–protein interaction network. Sixteen putative targets among the hub genes were conserved in all strains of *Leptospira*. Out of sixteen, fourteen were enzymes while 8 were novel and 4 were involved in virulence mechanism. In addition, genome scale metabolic network reconstruction and choke point analysis revealed cobA (porphyrin and chlorophyll metabolism) and thiL (thiamine metabolism) as chokepoints in their respective metabolic pathways. The proposed hub genes could act as putative broad-spectrum drug targets for *Leptospira* species, however, these putative targets should be validated to ensure them as real one prior to utilizing them for target based lead discovery.

## Introduction

The globally widespread occurrence of bacterial resistance to present drugs has stressed the necessity to find novel targets [1]. Human leptospirosis is a zoonotic infection caused by pathogenic spirochete *Leptospira* with the prevalence of about one million cases per year of which 60,000 failed to survive annually [2–4]. It is an endemic, occupational as well as recreational disease in tropical and rural areas [5–7]. Humans, who are exposed frequently to the diseased rodents, pet animals and polluted water, are at high risk of leptospirosis [8]. Clinical symptoms of the disease range from mild fever, vomiting, flu like illness, headache, diarrhea and muscle ache to multi-organ system complications which include kidney, liver, central nervous system and lungs with death rates 5% to 40% [9–10].

*Leptospira* infection cases have been highly recorded from Indonesia, Thailand, India, Sri Lanka and Maldives. India has been recognized as a major hub for *Leptospira* spp. to cause leptospirosis since 20^th^ century as it is a confluence of environmental, socio-economic and demographic factors [11]. Climate changes, population size, global warming, natural calamities (cyclone, floods), lack of facilities, poor sanitary infrastructure and clinical suspicion *etc*. increase the incidence of leptospirosis in coastal and rainy states of India like Gujarat, Kerala, Tamil Nadu, Maharashtra and Andaman-Nicobar islands [8,11–13].Various studies conducted in past few years confirmed higher prevalence of leptospirosis. Most substantial outbreaks have been observed in Chennai floods in 2015, spontaneous eruptions in Gujarat in 2011, flash floods in Mumbai in 2005 and cyclone in Orissa in 1999.

Taylor & Goyal *et al*., 1931, isolated *L*. *andamans* and *L*. *grippotyphosa* from diseased patients [14]. In 1960, Dalal P. M. provided the evidence of *L*. *icterohaemorrhagiae* antigen in jaundice patients. Similar report was submitted by Joseph K. M. in 1983 who found *Leptospira* infection in patients of jaundice. Muthusethupathi and Shivkumar observed renal failure in patients of Madras due to Leptospirosis. In 1996, Saravanan & Rajendran isolated *L*. *javanica* from urine sample of renal failure, cases of Chennai. Gujarat reported 130 deaths in 2011 within a span of two months due to leptospirosis. Recently, Kochi and Kerala reported 209 cases with 12 deaths. In October 2012, Gujarat reported 16 deaths. These reports highlights the continuous alarming jeopardy the disease presents at this hour.

Presently, treatment of severe leptospirosis is still unclear [15]. Antibiotics (penicillin, cephalosporins, azithromycin, doxycycline) and vaccines were relatively unsuccessful against *Leptospira* spp. This emphasizes the need of a new drug targets for evolution of competent drug that kill the pathogen [16–18]. In the last two decades, classical research approach was being used to classify protein targets towards the expansion of subunit and recombinant vaccines against leptospirosis. In 2005, whole genome analysis was carried out to classify potential vaccine against *L*. *Interrogans* [19]. Amineni *et al*., 2010 proposed 88 putative drug targets for two serovars of *L*. *interrogans* [20]. Lack of data, information as well as methodology, restrict the development of novel potent targets. Previous studies lack the gaps or information that would be useful in revealing of potent target like (1) coverage of all pathogenic strains, (2) metabolic reconstruction and system biology analysis, (3) exploration of hub genes that are analogous for endurance and virulence of pathogen, (4) recognition of choke point enzymes or reactions and (5) common target among the pathogenic strains.

In this study, we have tried to design a protocol to overcome the limitations of previous studies. Our protocol includes complete genome analysis, subtractive approach, comparative approach, and protein-protein interaction analysis that ultimately link to metabolomics. Various bioinformatics tools and cheminformatics techniques represent an attractive source of alternative method for target identification. The drug targets could be used, sequentially, for optimization and new lead recognition through free energy calculation, molecular modeling, molecular dynamics simulations, multiple docking strategies and drug-likeliness determination to establish new antibacterial agent against leptospirosis.

## Materials and methods

**Fig 1:**
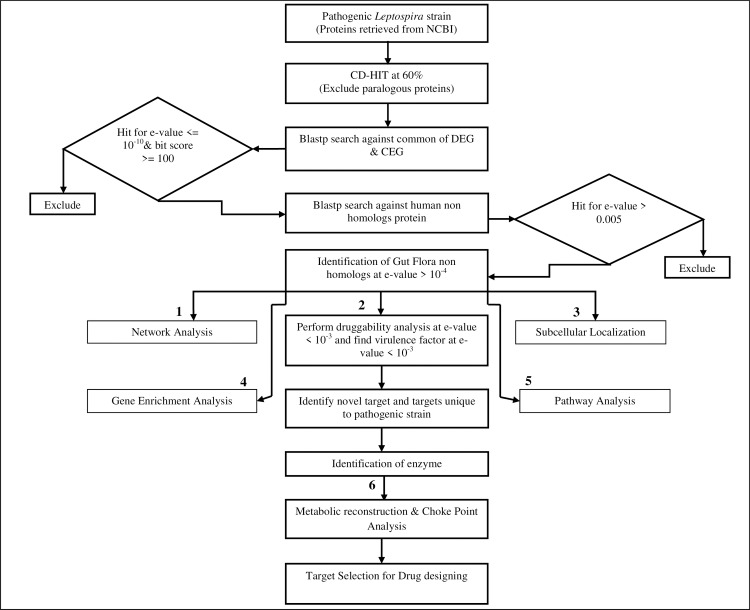
The complete protocol of the target identification and prioritization of pathogenic strains of *Leptospira*.

Pathogenic strains of *Leptospira* were subjected to a broad-spectrum anti-leptospiral target identification strategy that involves subtractive and comparative genomics approach along with protein-protein interaction network analysis. The complete protocol of the target identification is shown in Fig 1.

### Classification of *Leptospira* strains

Up to 2016, global character was lacking in classification of *Leptospira*. Fouts *et al* (2016) and Caimi *et al* (2017) have established the classification of *Leptospira* by wide range inter-species genomic comparison of all known infectious and non-infectious species of the bacterium [21–22]. In the present study, we have followed the systems established by Fouts and Caimi to classify various species of *Leptospira* as pathogenic, intermediate and saprophytic to humans. List of classified *Leptospira* strains was prepared and target mining using subtractive genomics, comparative genomics along with protein-protein interaction network analysis was restricted to pathogenic strains only.

### Mining of potential drug targets

Whole genome of pathogenic strains was retrieved and processed using target identification software called TiD developed by our team [23]. TiD is a standalone program. It consists of modules for paralog analysis, non-homolog analysis and essentiality analysis that exclude duplicates proteins, homolog of human and its gut flora proteins from the essential protein dataset [24–26]. Target prioritization module annotates the screened essential protein dataset on druggability and virulence which is essential for pathogen survival as well as pathogenicity [27].

Target identification and prioritization parameters were adapted from our previously published paper [23]. The standard protocol for target identification was used to remove paralogs from pathogenic leptospiral proteome and, then, define the list of essential genes that are homologs to entries present in Database of Essential Genes (DEG) with e-value ≤ 10^−10^, bit score ≥ 100. Further, the proteins homology was checked with human and gut microbes. The resulting dataset was compiled as putative drug targets and annotated for virulence, drug likeliness and mapped with UniProt identifiers (http://www.uniprot.org/).

### Metabolic pathway analysis

The mapped dataset of putative targets was prioritized at the KAAS (KEGG Automatic Annotation Server) to know the involvement of these proteins in metabolic pathways. Functional annotation of these targets was also acquired through BLAST comparisons against KEGG database [28]. KAAS server has programmed procedure that depends on sequence similarities and bi-directional best hit data to assign K numbers that permits recreation of KEGG pathways.

### Subcellular localization

Subcellular localization along with the biological significance of inimitable drug targets was analyzed in CELLO v.2.5. It is a multi-class support vector machine sorting method. This identifies targets as membrane proteins, cellular proteins or surface protein [29]. Results obtained were further analyzed in subcellular localization prediction tool pSORTb v.3.0. It is first standalone software that predicts the location of proteins for all prokaryotes including archaea and bacteria with atypical membrane/cell wall topologies with high accuracy [30].

### Genome scale metabolic network reconstruction

The metabolic network reconstruction of *L*. *borgpetersenii* serovar Hardjo-bovis str. L550 and *L*. *interrogans* serovar Copenhageni str. Fiocruz L1-130 were performed using ‘PathoLogic’ plugins of pathway tools [31]. GenBank file format of each chromosome of organism were used as input in ‘Build’ section that involved ‘Replicon Editor’, ‘Trial Parse’, ‘Automated Build’ and Refine. Pathway hole filler was used to fill the gaps of reconstructed network and then, save the database for choke point analysis.

### Choke-point analysis

"Chokepoint responses" are the reactions which exclusively catalyze a particular substrate or preferentially deliver a particular product [32–33]. To distinguish potential drug targets, we searched choke points reactions using Pathway tool. Choke point analysis includes all reactions that raise several pathways while excludes those reactions catalyzed by more than one enzyme and found in human. Result of choke point analysis was compared with putative targets. Results of Pathway tools were also validated with BioCyc webserver [34]. It is an online server that comprised of 13075 pathway genome databases and provides unified data on the genomes and metabolic pathways of thousands of sequenced organisms which are built *via* metabolic network reconstruction software(s).

### Sequence-structure relationship

A multiple sequence alignment was created for cobA and thiL sequences of *Leptospira* strains using clustalX and UniProt. Structurally conserved elements were identified and formatted with ESPript 3.0 to obtain the conserved and semi conserved residues of both the chokepoints [35]. The secondary structure of cobA and thiL were determined by using PsiPred server. Further, a three dimensional structure model of a target sequences were built by Modeller 9.17 [36]. Energy minimization of best modelled structure having least DOPE score was performed using YASARA server [37]. Validation of energy minimized structures was performed through SAVES server ((http://services.mbi.ucla.edu/SAVES/), QMEAN and ProSA [38–39]. The catalytic pocket and active site residues of validated model were defined using CASTp and PyMOL-2.3.2 [40–41].

### Protein-protein network analysis

Protein-protein interaction data is available for two strains of *Leptospira* in string database. Therefore, network analysis was restricted to these two strains. PPI network of putative drug targets of both strains were constructed in STRING app of Cytoscape v3.7.1 with confidence score ≥0.4 [42]. Network interaction data was figured through network analyzer module [43]. Putative drug targets with interacting partners’ ≥30 in *Leptospira* proteome were subjected to MCODE plugin for the functional module detection [44]. The cutoff parameters used for molecular complex detection were degree cutoff = 2, node score cutoff = 0.2, k-core = 2, and maximum depth = 100. The highest ranked module was chosen for gene ontology and functionality. Gene enrichment analysis of interacting nodes was also carried in STRING app. The results of two strains were checked for conservancy among the pathogenic strains using multiple sequence alignment tool ClustalX2 [45].

## Results and discussion

The current study is an adoption of advanced subtractive and comparative genomic approach and further augmented to protein-protein interaction network and metabolomics analysis. The unique and essential proteins are significant for *Leptospira* growth, survival and pathogenicity. A search for the hub proteins and choke enzymes in the unique and essential pathways is therefore considered as a promising approach to deal with the challenging leptospirosis infection.

### Classification of *Leptospira* strain

*In silico* and *in vitro* target identification research to cure leptospirosis, till date, have been more focused towards most pathogenic *L*. *interrogans* serovars named Lai and Copenhagani [20,46–47]. The present study tried to cover all pathogenic strains of *Leptospira* which are responsible for causing infectious disease. We grouped *Leptospira* strains based on their pathogenicity. Out of 27 retrieved strains from NCBI FTP server, 16 are observed to be pathogenic in nature, 5 are intermediate and 6 are saprophytic in nature (Table 1). Sixteen pathogenic strains of *Leptospira* were selected for putative drug target mining [21–22,48].

**Table 1:** List of *Leptospira* strains and their pathogenicity.

S. No.	*Leptospira* strain	Accession ID	Pathogenicity
**1**	*Leptospira alexanderi* serovar Manhao 3 str. L 60	GCA_000243815.3_gls454062v02	Pathogen
**2**	*Leptospira* alstonii	GCA_001729245.1_ASM172924v1	Pathogen
**3**	*Leptospira borgpetersenii* serovar Hardjo-bovis str. L550	GCA_000013945.1_ASM1394v1	Pathogen
**4**	*Leptospira interrogans* serovar Copenhageni str. Fiocruz L1-130	GCA_000007685.1_ASM768v1	Pathogen
**5**	*Leptospira kirschneri* serovar Cynopteri str. 3522 CT	GCA_000243695.3_gls454049v02	Pathogen
**6**	*Leptospira kmetyi* serovar Malaysia str. Bejo-Iso9	GCA_000243735.3_gls454052v1.0	Pathogen
**7**	*Leptospira mayottensis* 200901122	GCA_000306335.2_gls454125v02	Pathogen
**8**	*Leptospira noguchii* serovar Panama str. CZ214	GCA_000306255.2_gls454059v02	Pathogen
**9**	*Leptospira santarosai* serovar Shermani str. LT 821	GCA_000313175.2_ASM31317v2	Pathogen
**10**	*Leptospira* sp. B5-022	GCA_000347035.1_gls454192v01	Pathogen
**11**	*Leptospira* sp. Fiocruz LV3954	GCA_000306435.2_gls454068v2.0	Pathogen
**12**	*Leptospira* sp. Fiocruz LV4135	GCA_000346675.1_gls454076v02	Pathogen
**13**	*Leptospira* sp. P2653	GCA_000346955.1_gls454051v01	Pathogen
**14**	*Leptospira* sp. ZV016	GCA_001584255.1_ASM158425v1	Pathogen
**15**	*Leptospira* sp. serovar Kenya str. Sh9	GCA_000347195.1_gls454066v01	Pathogen
**16**	*Leptospira weilii* serovar Topaz str. LT2116	GCA_000244815.3_gls454188v02	Pathogen
**17**	*Leptospira* sp. CLM-U50	GCA_002150035.1_ASM215003v1	Pathogenic
**18**	*Leptospira meyeri*	GCA_000304275.1_gls454017v1.0	Saprophytic
**19**	*Leptospira terpstrae*	GCA_000332495.2_gls454203v02	Saprophytic
**20**	*Leptospira vanthielii*	GCA_000332455.2_gls454199v02	Saprophytic
**21**	*Leptospira wolbachii*	GCA_000332515.2_gls454195v02	Saprophytic
**22**	*Leptospira yanagawae*	GCA_000332475.2_gls454202v02	Saprophytic
**23**	*Leptospira biflexa*	GCA_000017685.1_ASM1768v1	Saprophytic
**24**	*Leptospira fainiei*	GCA_000306235.2_gls454058v2.0	Intermediate
**25**	*Leptospira inadai*	GCA_000243675.3_gls454047v02	Intermediate
**26**	*Leptospira licerasiae*	GCA_000244755.3_ASM24475v3	Intermediate
**27**	*Leptospira wolfii*	GCA_000306115.2_gls454061v02	Intermediate
**28**	*Leptospira bromii*	GCA_000243715.3_gls454050v02	Intermediate

### Sequence retrieval and putative drug targets mining

**Fig 2:**
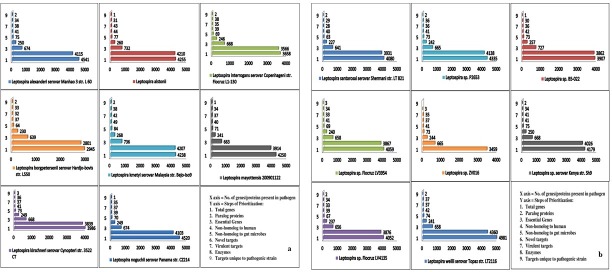
Sequence retrieval and putative drug targets mining. The plot of detailed protein products of *L*. *alexandari*, *L*. *alstonii*, *L*. *borgpetersenii*, *L*. *interrogans*, *L*. *kirschneri*, *L*. *kmetyi*, *L*. *mayottensis*, *L*. *noguchii* is represented in Fig 2A. The plot of detailed protein products of *L*. *sp*. B5-022, *L*. *sp*. Fiocruz LV3954, *L*. *sp*. Fiocruz LV4135, *L*. *sp*. P2653, *L*. *sp*. ZV016, *L*. *sp*. serovar Kenya str. Sh9, *L*. *santarosai* serovar Shermani str. LT 821, *L*. *weilii* serovar Topaz str. LT2116 is represented in Fig 2B. The resulted drug targets in each step represented using bar graph.

Complete amino acid sequence (*.faa) of sixteen serovars were successfully retrieved from NCBI-FTP server and subjected to TiD software. Nascimento *et al*., 2004 and Ren *et al*., 2003 reported the genomic size of *L*. *interrogans i*.*e*. to be 4.33-Mb chromosome I and 350-kb chromosome [49–50]. In the present study, *L*. *weilii* serovar Topaz str. LT2116 proteome consists of most elevated number of proteins while *L*. *borgpetersenii* serovar Hardjo-bovis str. L550 has smallest proteome of 2945 proteins. About 13 pathogenic *Leptospira* strains have larger proteome than *L*. *interrogans*. Size of each proteome is mentioned in Fig 2A and 2B. Amineni *et al*., 2010 mentioned 158 essential genes of *Lai* and 218 genes of *Copenhagni* as human non homolog [20]. In our study, 736 proteins of *L*. *kmetyi* serovar Malaysia str. Bejo-Iso9 followed by 732 proteins of *L*. *alstonii* are identified as essential proteins. *L*. *borgpetersenii* serovar Hardjo-bovis str. L550 has lowest number of essential proteins (Fig 2A and 2B). *L*. *kmetyi* serovar Malaysia str. Bejo-Iso9 has uppermost whereas *L*. *santarosai* serovar Shermani str. LT 821 has minimum number of human non-homolog as well as gut flora non homolog protein. All strains of *Leptospira* have more than 36 novel drug targets and 31 virulent proteins. Results of drug target mining of each pathogenic strain are reported in Fig 2A and 2B.

### Metabolic pathway analysis

**Fig 3:**
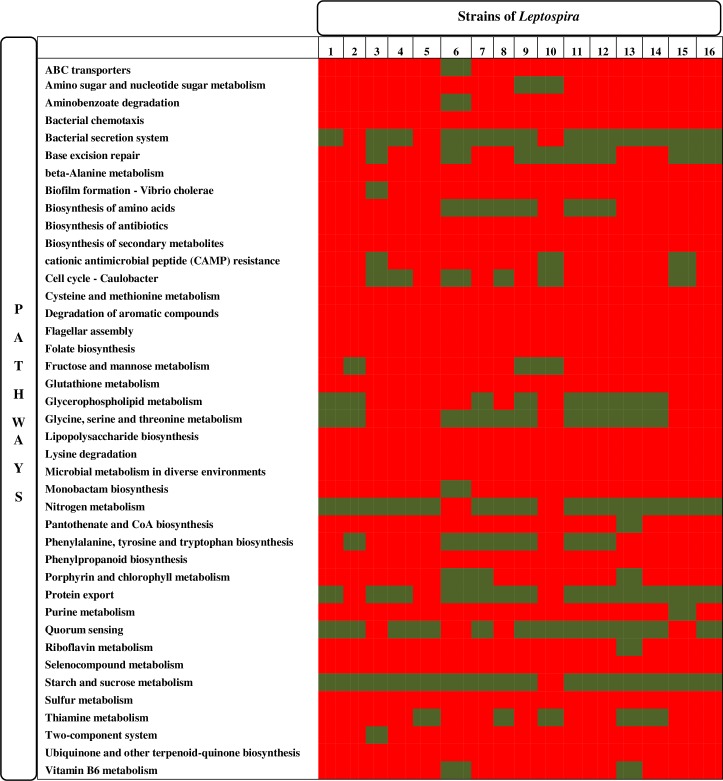
Metabolic pathways of the host and pathogen. Sixteen pathways are unique to all strains of *Leptospira* to which 34 common genes were retrieved as putative drug targets. Red color denotes presence while Green color denotes absence of pathways from which common genes were retrieved for further analysis.

Metabolic pathway analysis of the host-pathogen highlighted sixteen pathogen specific pathways such as biosynthesis of antibiotics, biosynthesis of secondary metabolites, degradation of aromatic compounds, microbial metabolism in diverse environments, sulfur metabolism, cysteine and methionine metabolism, seleno compound metabolism, lysine degradation, beta-alanine metabolism, glutathione metabolism, folate biosynthesis, lipopolysaccharide biosynthesis, ubiquinone and other terpenoid-quinone biosynthesis, phenylpropanoid biosynthesis, bacterial chemotaxis and flagellar assembly. These pathways were observed to have 34 communal drug targets (Fig 3, Table 2). In addition, common drug targets were also identified from 8 pathways which are unique to the survival of 15 strains of *Leptospira*. The findings of our study are in same line with the Amineni *et al*., 2010 and Anisetty *et al*. 2005 [20,51]. Among these common drug targets three are non enzyme and 31 are enzymes (Table 2).

**Table 2:** List of common drug target extracted from the shared pathways of 16 *Leptospira* strains.

S. No	Gene Product	Description	Enzyme/Non-Enzyme	Subcellular Localization	Choke Point
**1**	pdxA	Pyridoxal phosphate biosynthesis	Enzyme	Cytoplasmic	NA
**2**	cysH	Phosphodenosine phosphosulphate reductase	Enzyme	Cytoplasmic	NA
**3**	Sir2	NAD-dependent protein deacylase	Enzyme	Cytoplasmic	NA
**4**	panB	3-methyl-2-oxobutanoate hydoxymethyltranferase	Enzyme	Cytoplasmic	NA
**5**	metX	Homoserine O-acetyltransferase	Enzyme	Cytoplasmic	NA
**6**	lpxA	acyl-[acyl-carrier-protein]—UDP-N-acetylglucosamine O-acyltransferase	Enzyme	Cytoplasmic	NA
**7**	lpxB	Lipid-A-disaccharide synthase	Enzyme	Outer membrane	NA
**8**	ribE	6,7-dimethyl-8-ribityllumazine synthase	Enzyme	Cytoplasmic	NA
**9**	lpxK	Tetraacyldisaccharide 4’-kinase	Enzyme	Outer membrane	NA
**10**	thiL	thiamine-phosphate kinase	Enzyme	Cytoplasmic	Choke Point
**11**	cysD	Sulfate adenylyltransferase subunit 2	Enzyme	Cytoplasmic	NA
**12**	kdsB	3-deoxy-manno-octulosonate cytidylyltransferase	Enzyme	Cytoplasmic	NA
**13**	panD	aspartate alpha-decarboxylase	Enzyme	Cytoplasmic	NA
**14**	wecB	UDP-N-acetylglucosamine2-epimerase	Enzyme	Cytoplasmic	NA
**15**	manA	Mannose-6-phosphate isomerase	Enzyme	Cytoplasmic	NA
**16**	trpF	N-(5'-phosphoribosyl)anthranilate isomerase	Enzyme	Cytoplasmic	NA
**17**	panC	Pantoate-beta-alanine ligase	Enzyme	Cytoplasmic	NA
**18**	gshA	Glutamate-cysteine ligase	Enzyme	Cytoplasmic	NA
**19**	kdtA	3-deoxy-D-manno-octulosonic-acid transferase	Enzyme	Transmembrane	NA
**20**	lpxC	UDP-3-O-[3-hydroxymyristoyl]N-acetylglucosamine deacetylase	Enzyme	Cytoplasmic	NA
**21**	lpxD	UDP-3-O-(3-hydroxymyristoyl)glucosamine N-acyltransferase	Enzyme	Cytoplasmic	NA
**22**	ubiA	4-hydroxybenzoate octaprenyltransferase	Enzyme	Transmembrane	NA
**23**	ubiX	3-polyprenyl-4-hydroxybenzoate decarboxylase	Enzyme	Cytoplasmic	NA
**24**	gmhA	Phosphoheptose isomerase	Enzyme	Cytoplasmic	NA
**25**	pdxJ	pyridoxine 5'-phosphate synthase	Enzyme	Cytoplasmic	NA
**26**	glmU	UDP-N-acetylglucosamine diphosphorylase	Enzyme	Cytoplasmic	NA
**27**	queF	NADPH-dependent 7-cyano-7-deazaguanine reductase	Enzyme	Cytoplasmic	NA
**28**	cobA	Cob(l)alaminadenosyltransferase	Enzyme	Cytoplasmic	Choke Point
**29**	mqnC	Dehypoxanthinefutalosinecyclase	Enzyme	Cytoplasmic	NA
**30**	kamA	L-lysine 2,3-aminomutase	Enzyme	Cytoplasmic	NA
**31**	cheR	Methylase of chemotaxis methyl-accepting protein	Enzyme	Cytoplasmic	NA
**32**	fliN	Flagellar motor switch protein	Non- Enzyme	Transmembrane	NA
**33**	mcp	MCP methylation inhibitor	Non- Enzyme	Transmembrane	NA
**34**	cheA	Two-component system sensor histidine kinase	Non- Enzyme	Transmembrane	NA

### Subcellular localization

Localization of 34 common drug targets in current study exposed that 29 drug targets are cytoplasmic and 5 are transmembrane proteins (Table 2). The membrane proteins have the capability to act as useful vaccine drug targets against all strains of *Leptospira*. However, these transmembrane proteins are involved in lipid polysaccharide synthesis (kdtA), ubiquinone-terpenoid-quinone biosynthesis (ubiA), bacterial chemotaxis (fliN, cheA, mcp)as well as part of flagellar assembly (fliN) and two component system (mcp, cheA). These play an essential role in the survival and pathogenesis of *Leptospira*. Location of these drug targets is required in future to design drug or vaccine accordingly. Subcellular localization information of a drug target should complement with the pharmacological properties of lead molecules targeted to it, therefore, it is an important aspect in rational drug design.

### Genome scale metabolic network reconstruction

Genome scale metabolic network of *L*. *borgpetersenii* serovar Hardjo-bovis str. L550 and *L*. *interrogans* serovar Copenhageni str. Fiocruz L1-130 were constructed successfully using Pathway Tools Software. Model of *L*. *interrogans* serovar Copenhageni str. Fiocruz L1-130 comprised 673 enzymes, 988 enzymatic reactions, 6 transport reactions, 133 pathways, 957 compounds, 37 tRNAs, 6 transporter and 3684 polypeptides (S1 SBML). Likewise, model of *L*. *borgpetersenii* serovar Hardjo-bovis str. L550 constructed with 141 pathways, 613 enzymes, 1099 enzymatic reactions, 5 transport reactions, 3290 polypeptides, 12 transporters and 1075 metabolites (S2 SBML). From the set of 34 drug target, 20 and 23 were shaped in reconstructed model of *L*. *borgpetersenii* serovar Hardjo-bovis str. L550 and *L*. *interrogans* serovar Copenhageni str. Fiocruz L1-130 respectively. These reconstructed models were used for choke point analysis.

### Choke point analysis

Chokepoint enzymes are enzymes that catalyze a reaction which either uniquely consume a substrate or interestingly deliver a precise product. If an enzyme catalyzes at least one chokepoint reaction, it is classified as a promising drug target. Accordingly, we expect the hindrance of a protein that expends an exceptional substrate brings aggregation in the remarkable substrate. This may be conceivably harmful to the cell and restraint the compound that delivers one of a kind item which further leads to the starvation of special product [32–33].

Pathway tool generated the report of choke point reactions for reconstructed model of *L*. *interrogans* serovar Copenhageni str. Fiocruz L1-130 and *L*. *borgpetersenii* serovar Hardjo-bovis str. L550. The report consists of choke point reactions on the consuming side as well as producing side. Target cobA (cob(I)yrinic acid a) and thiL (thiamine-phosphate kinase) is found as a choke enzyme in both the reconstructed genome scale metabolic model of Leptospiral strains (Table 2). The cobA with an EC: 2.5.1.17 participated in cobalamin biosynthesis pathway. Pathogenic strains of *Leptospira* can’t grow in the absence of cobalamin that’s why it is an essential component of the Ellinghausen-McCullough-Johnson-Harris (EMJH) semi-synthetic selective medium [49–50]. In contrast, gene thiL found to be important in conversion of thiamine monophosphate to thiamine pyrophosphate which is essential for pathogen survival [52]. Results of pathway tool were also validated with BioCyc server that shows cobA as choke point in most of the Leptospiral strain and thiL in *L*. *mayottensis* 200901122 and *L*. *kmetyi* serovar Malaysia str. Bejo-Iso9.

### Sequence alignment, homology modelling and validation

**Fig 4:**
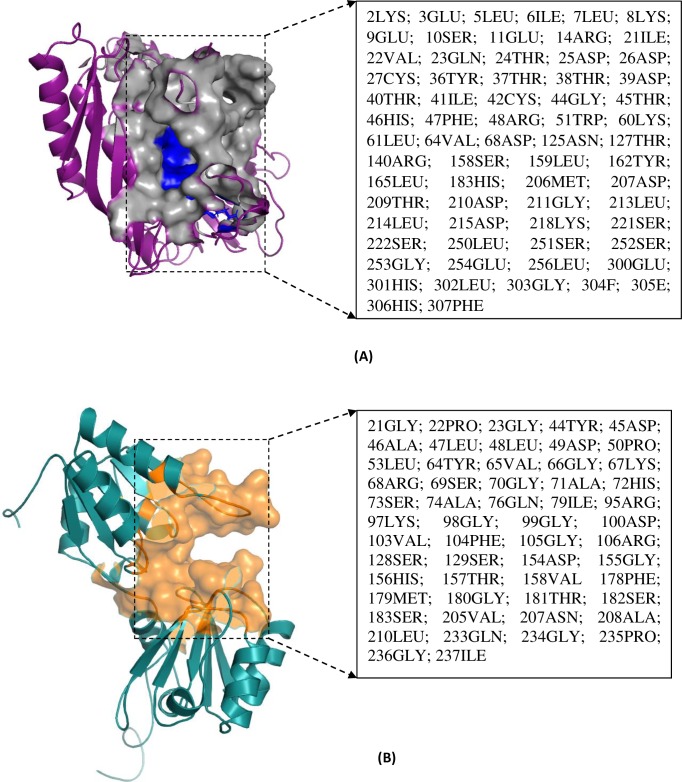
Active site prediction of thiL and cobA. (A) Largest binding site of modelled structure of thiL (magenta color) showing the active site cleft (grey color). Key residues of binding pocket are highlighted with blue color while rests are shown in a box. (B) Likewise, surface view of binding pocket of cobA (green color) is very deep in comparison with thiL. Binding residues present in largest pocket of modelled protein is presented in orange color.

Multiple sequence alignment was performed for cobA and thiL gene within the pathogenic *Leptospira* strain (Table 1). The result showed conserved, semi conserved and dispersed amino acid residues. The semi-conserved residues were showen with dot and conserved with red star (S1 and S2 Files). ESPript defined MSA of cobA showed the 10 αhelix (9α + η), 10 β-sheets and 6 turns (TT) while 9 αhelix (8α + η), 12 β-sheets and 7 turns (TT) were found in thiL. In addition, psiPred server validated the ESPript report for both the chokepoints. All these results were in agreement with each other for the secondary structure elements of cobA and thiL. Afterwards, 3D structure of thiL and cobA were modelled using 2YBQ_A (Query Coverage: 88%, Identity: 39.36%) and 3C9R_A (Query Coverage: 88%, Identity: 30.32%) as template respectively (Fig 4). The quality of the modelled structures after energy minimization was evaluated on the UCLA SAVES server shown in Table 3. CastP predicted the largest binding pocket of both modelled structures. Surface view of cobA is showing a deep binding pocket while thiL represented Gln23, Thr24, Asp25, Asp26, Asp39, Asp68, Arg140, Asp207, Thr209 and Asp210 as key residues in their binding pocket (Fig 4).

**Table 3:** Refinement and quality assessment of modelled structure of thiL and cobA.

	Verify3D	ERRAT	PROVE	PROCHECK			Prosa Z Score	QMEAN
				Favoured	Allowed	Disallowed		
**thiL**	86.38% of the residues have averaged 3D-1D score > = 0.2 Pass	84.1549	55 buried outlier protein atoms, 5.3% (Error)	289 (94.8%)	13 (4.3%)	3 (1.0%)	-7.8	-2.26
**cobA**	85.02% of the residues have averaged 3D-1D score > = 0.2 Pass	95.935	2 7 buried outlier protein atoms, 3.4% (Error)	269 (97.1%)	7 (2.5%)	3 (1.0%)	1 (0.4%)	-1.24

### Prioritization of drug targets

**Fig 5:**
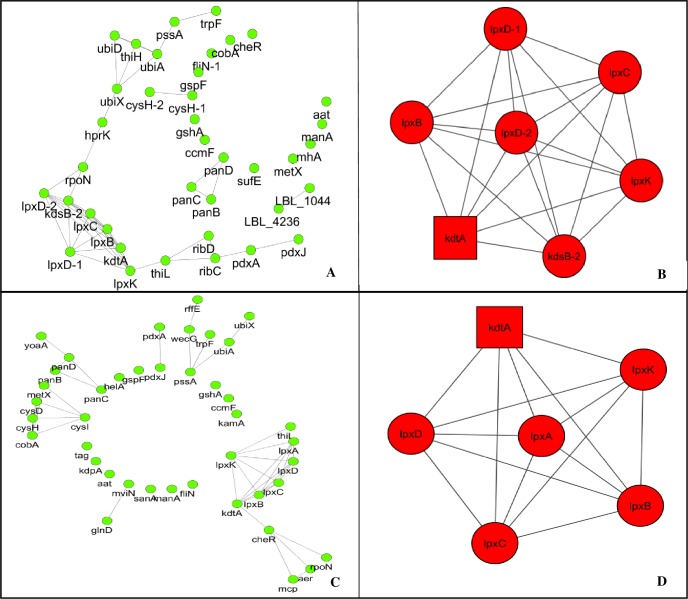
Network analysis and molecular function detection. (A) & (B) present the protein-protein interaction within the selected proteins of *L*. *borgpetersenii* serovar Hardjo-bovis str. L550 whereas (C) & (D) denote the interacting partners of *L*. *interrogans* serovar Copenhageni str. Fiocruz L1-130. In PPI network, nodes denote protein and interaction between the 2 nodes denotes the edge. Significant MCODE cluster (B) and (D) denotes the genes which are involved in polysaccharide biosynthesis pathway.

Putative drug targets were subjected for prioritization through network analysis, molecular complex detection and gene enrichment analysis (Fig 1). As mentioned, string app is restricted to generate protein-protein interaction network of *L*. *borgpetersenii* serovar Hardjo-bovis str. L550 and *L*. *interrogans* serovar Copenhageni str. Fiocruz L1-130. Network of *L*. *borgpetersenii* comprised of 38 nodes with 41 edges. Gene lpxD-2, kdsB-2 and lpxK demonstrated the most noteworthy degree followed by kdtA, lpxD-1, lpxB and lpxC with degree 6 (Table 4; Fig 5A). Similarly, *L*. *interrogans* Copenhageni L1-130 had 41 nodes with 40 edges. Most astounding degree hub is observed for kdtA and lpxK followed by lpxD, lpxB, lpxC and lpxA (Table 5; Fig 5C). Extension of these two networks, with all proteins of pathogen, stretched the estimation of best genes of the system and predicts with assurance the essential role of these genes for pathogen (Tables 4 and 5). After network extension, degree of cheR increased from 0 to 91, fliN from 0 to 68, cobA from 0 to 35 and metX from 0 to 38 in case of *L*. *borgpetersenii* Hardjo-bovis L550 while degree of cheR increased from 4 to 76, fliN from 0 to 66, cob A from 4 to 47 and metX from 2 to 39 in case *L*. *interrogans* Copenhageni L1-130.

**Table 4:** List of Interacting partners within selected proteins as well as within all proteins of *L*. *borgpetersenii serovar Hardjo-bovis str*. *L550*.

Proteins	Description	Interaction_selected Proteins	Interaction_All Proteins of Pathogen
lpxK	Tetraacyldisaccharide 4'-kinase	7	79
kdsB-2	3-deoxy-manno-octulosonate cytidylyltransferase	7	55
lpxD-2	UDP-3-O-[3-hydroxymyristoyl] glucosamine N-acyltransferase	7	51
lpxC	UDP-3-O-[3-hydroxymyristoyl] N-acetylglucosamine deacetylase	6	60
lpxB	Lipid-A-disaccharide synthase	6	76
lpxD-1	UDP-3-O-[3-hydroxymyristoyl] glucosamine N-acyltransferase	6	73
kdtA	3-deoxy-D-manno-octulosonic-acid (KDO) transferase	6	79
ubiA	Prenyltransferase	4	46
ubiX	3-polyprenyl-4-hydroxybenzoate decarboxylase	4	51
ubiD	3-polyprenyl-4-hydroxybenzoate decarboxylase	3	34
ribC	Riboflavin synthase subunit alpha	3	17
rpoN	DNA-directed RNA polymerase sigma-54 subunit	3	71
thiH	Thiamine biosynthesis enzyme	3	41
thiL	Thiamine monophosphate kinase	3	23
panD	Aspartate alpha-decarboxylase	2	44
panC	Pantoate—beta-alanine ligase	2	27
panB	3-methyl-2-oxobutanoate hydroxymethyltransferase	2	60
ribD	pyrimidine deaminase, riboflavin biosynthesis	2	56
hprK	HPr kinase/phosphorylase	2	39
pdxA	4-hydroxythreonine-4-phosphate dehydrogenase	2	66
pssA	CDP-diacylglycerol—serine O-phosphatidyltransferase	2	41
LBL_4236	Lysine 2,3-aminomutase	1	39
LBL_1044	Lysine 2,3-aminomutase	1	39
pdxJ	Pyridoxine 5'-phosphate synthase	1	50
trpF	Phosphoribosylanthranilate isomerase	1	42
cysH-2	Sulfate adenylyltransferase subunit 2	1	14
cysH-1	Phosphoadenylyl-sulfate reductase (thioredoxin)	1	13
sufE	Fe-S metabolism protein	0	38
ccmF	Cytochrome c biogenesis protein	0	49
gshA	Gamma-glutamylcysteinesynthetase	0	25
gspF	Type II secretory pathway component, protein F	0	21
fliN-1	Endoflagellar motor switch protein	0	91
cobA	Cob(I)yrinic acid a,c-diamideadenosyltransferase	0	35
cheR	Methyltransferase of chemotaxis protein	0	68
aat	Leucyl/phenylalanyl-tRNA—protein transferase	0	18
manA	Mannose-6-phosphate isomerase	0	18
gmhA	Phosphoheptose isomerase	0	17
metX	Homoserine O-acetyltransferase	0	11

**Table 5:** List of Interacting partners within selected proteins as well as within all proteins of *L*. *interrogans* serovar Copenhageni str. Fiocruz L1-130.

Proteins	Description	Interaction_selected Proteins	Interaction_All Proteins of Pathogen
kdtA	3-deoxy-d-manno-octulosonic acid transferase	6	109
lpxK	Tetraacyldisaccharide 4'-kinase	6	57
lpxD	UDP-3-O-[3-hydroxymyristoyl] glucosamine N-acyltransferase	5	70
lpxB	Lipid-a-disaccharide synthase protein	5	66
lpxC	UDP-3-O-[3-hydroxymyristoyl] N-acetylglucosamine deacetylase	5	70
lpxA	UDP-N-acetylglucosamine acyltransferase	5	76
cysI	Sulfite reductase subunit beta	4	40
cheR	Chemotaxis protein methyltransferase	4	76
cobA	Uroporphyrinogen-III C-methyltransferase	4	47
pssA	Phosphatidylserine synthase	3	37
cysH	Phosphoadenosine phosphosulphate reductase	3	32
cysD	Sulfate adenylyltransferase subunit 2	3	32
panD	Aspartate alpha-decarboxylase	3	36
aer	Chemotaxis protein	2	9
metX	Homoserine O-acetyltransferase	2	39
mcp	Chemotaxis protein	2	84
wecG	UDP-n-acetyl-d-mannosamine transferase	2	48
panB	3-methyl-2-oxobutanoate hydroxymethyltransferase	2	39
panC	Pantoate—beta-alanine ligase	2	66
ubiA	Prenyltransferase	2	50
thiL	Thiamine-monophosphate kinase protein	1	33
rpoN	RNA polymerase sigma-54 factor	1	119
pdxA	Pyridoxal phosphate biosynthesis protein	1	28
glnD	Protein-PII uridylyltransferase	1	28
mviN	hHypothetical protein	1	52
trpF	N-(5'-phosphoribosyl)anthranilate isomerase	1	44
rffE	UDP-N-acetylglucosamine 2-epimerase	1	106
ubiX	3-octaprenyl-4-hydroxybenzoate carboxy-lyase	1	30
yoaA	ATP-dependent helicase	1	58
pdxJ	Pyridoxine 5'-phosphate synthase	1	37
manA	Mannose-6-phosphate isomerase	0	24
sanA	Vancomycin resistance protein	0	3
aat	Leucyl/phenylalanyl-tRNA—protein transferase	0	24
kdpA	Potassium-transporting ATPase subunit A	0	21
tag	3-methyl-adenine DNA glycosylase I	0	4
helA	Heavy metal efflux pump	0	8
gspF	General secretory pathway protein F	0	20
gshA	Gamma-glutamylcysteine synthetase	0	11
ccmF	Cytochrome C biogenesis protein	0	18
kamA	L-lysine 2,3-aminomutase	0	12
fliN	Flagellar motor switch protein	0	66

Molecular code detection produces one significant cluster with MCODE score = 15.733 for *L*. *borgpetersenii* Hardjo-bovis L550. It comprised 16 hubs associated through 118 edges. All 16 hubs are similarly connecting with one another with the degree of 13 to 15. Gene kdtA is found to be the seed protein of PPI network (Fig 5B). Gene enrichment analysis displayed lpxD-2, kdsB2, lpxC, lpxB, kdtA, lpxK, lpxD-1 and manA. These are markedly enriched in positive regulation of lipopolysaccharide biosynthesis while most of the genes are involved in metabolic pathways. Similarly, *L*. *interrogans* Copenhageni Fiocruz L1-130 module 1 obtained MCODE score = 14.857 with 15 nodes that interconnected with 104 edges. Gene kdsB is the seed of network which is connected to 13 other genes of the network (Fig 5D). Gene enrichment displayed 4 genes fliN, cheR, mcp and aer. These genes are part of two component system and involved in bacterial chemotaxis. The gene ontology analysis indicated that selected essential genes of both the pathogens are important part of metabolic pathways and lipopolysaccharide biosynthesis (Fig 5). Extended network added ribB, ribC, cysl, cysH, cysD, cobA and cysG genes which are responsible for riboflavin biosynthesis, microbial metabolism in diverse environment, sulfur metabolism porhyrin and cholorophyll metabolism (Tables 4 and 5).

### Comparative analysis

**Fig 6:**
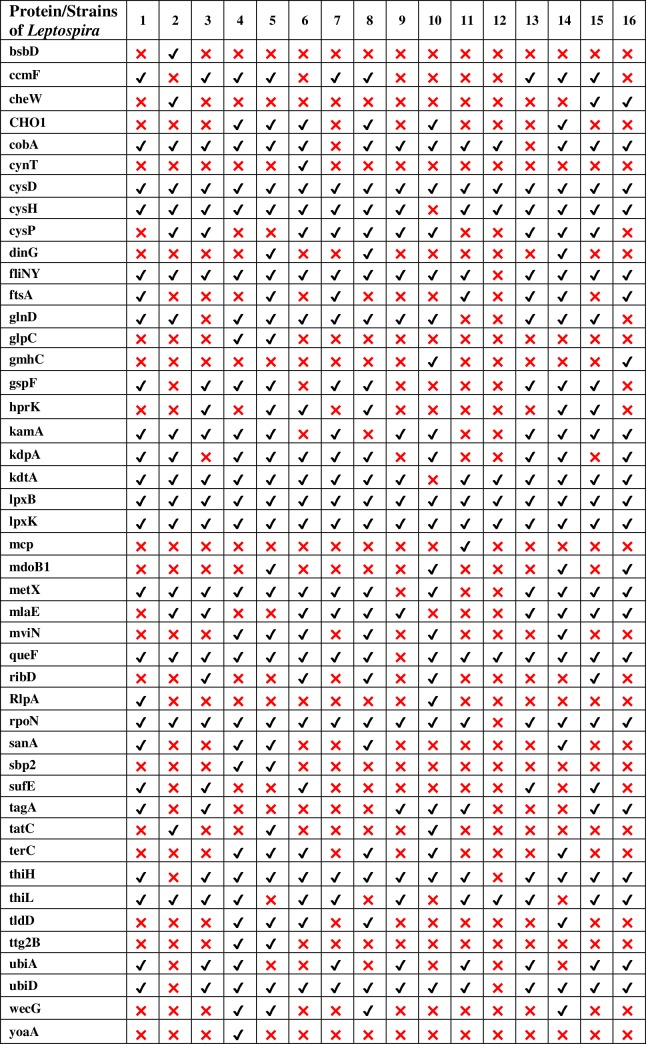
Druggability analysis of proteins of *Leptospira* strains. X axis consists of number of strains and Y axis contains non-homolog gut flora proteins present in *Leptospira* strains. Right mark denotes the presence of novel target in each strain. Gene product lpxK, lpxB and cysD found to be novel in all strains of pathogen whereas in 16 out of 15 strains, kdtA, fliN, rpoN and queF are seen to be novel.

**Fig 7:**
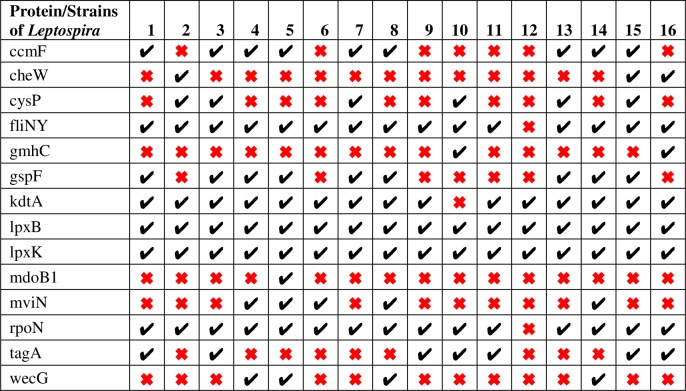
Virulent analysis of proteins of *Leptospira* strains. X axis consists of number of strains and Y axis contains non-homolog gut flora proteins present in *Leptospira* strains. Right mark denotes the presence of virulent target in each strain. Gene product lpxK and lpxB found to be virulent in all strains of pathogen whereas in 16 out of 15 strains, kdtA and rpoN are seen to be virulent. Protein fliN is important for virulence in 14 strains of *Leptospira*.

**Fig 8:**
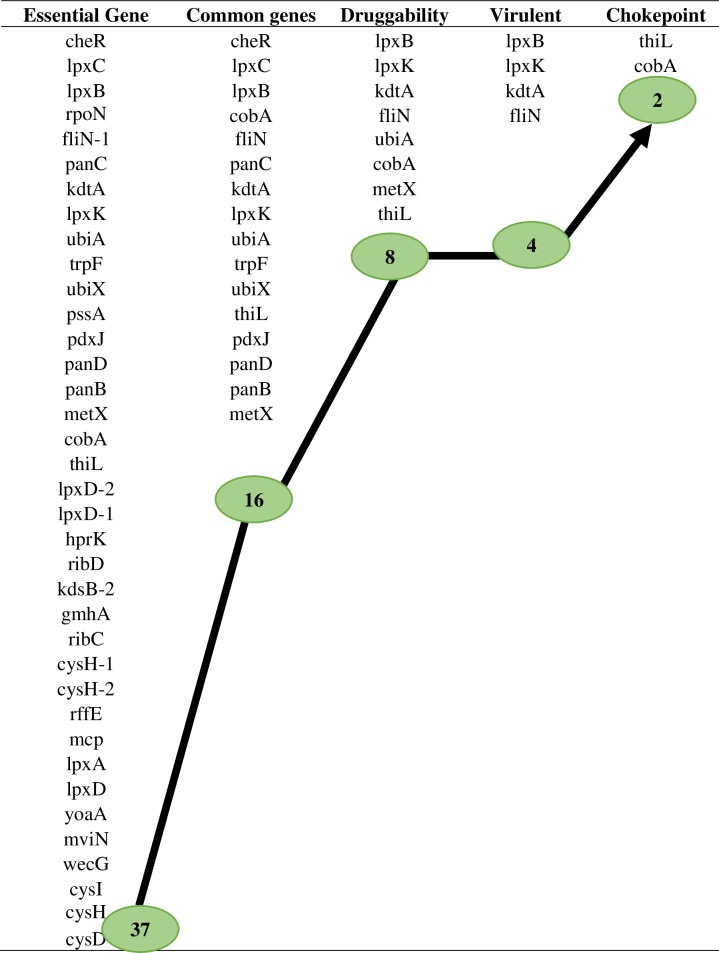
Comparative analysis of essential proteins. Comparative analysis revealed 37 genes are essential. Among these, 16 are common in all strains of *Leptospira* from which 8 are found to be novel. From novel targets of *Leptospira*, 2 are acting as choke points and fliN protein is virulent in nature.

Comparison between both the networks showed 37 putative targets having the degree ≥30 among which 18 hubs are observed to be common in both pathogens. Among the hub proteins, 16 are also shortlisted from the set of 34 common putative drug targets. Multiple sequence alignment revealed that these hub targets are conserved in at least 10 strains of pathogen. Among these common hub proteins, 8 are novel in at least 10 strains, 5 are virulent while 2 are novel as well as common drug targets (Figs 6 and 7). Hence, these 8 proteins lpxB, lpxK, kdtA, fliN, cobA, metX, thiL and ubiA are proposed as putative drug targets in the present study, as absence of these proteins would affect the survival and pathogenicity of pathogen. However, target proteins lpxB, lpxK, kdtA, cobA, metX and ubiA were previously reported in the study of Amineni *et al*., 2010 for 2 pathogenic strains of *Leptospira* through subtractive genomic analysis but fliN and thiL are novel targets which are reported first time in our study [20] (Fig 8).

The functional importance of the target candidates and the pathways involved are discussed here. As mentioned, proposed 8 drug targets majorly belong to 7 essential pathogen specific pathways. Amphipathic lipopolysaccharides are outer cell wall surface antigen of *Leptospira* and actively participate in virulence and antibiotic sensitivity [53–54]. Lipopolysaccharides also act as primary barrier of pathogen and maintain the osmolarity of the gram negative cell. Inhibitor targeting lpxB, lpxK and kdtA proteins of outer membrane would affect the formation of primary barrier and alter the osmotic environment of pathogenic strains. This could be helpful to cure the leptospiral infections successfully. Taylor *et al*., 2008 and Raetz *et al*., 2002 also reported the proteins of this pathway as an efficient drug targets in other bacterial pathogens [20,55–56].

One of the central and defining reasons of increasing prevalence rate of Leptospirosis is the capability of the pathogenic strains to switch their flagellar motility for highly effective translocation via viscous substrates and tissues that permits access to far-away host niches [57]. Flagellar motor protein consists of three proteins among which fliN is one of major protein of flagellar motor switch system present in the basal body and interacts with chemotaxis proteins to define the translational and rotational motion of flagella [49,50]. Liao *et al*., 2009 showed inactivation of fliN gene that affects the rotative motion and migration in liquid and semi-solid medium respectively [58]. Hence, these complex set of genes involved in endoflagellum and its basal rotor system formation play critical role in the motility and virulence to cause Leptospirosis.

Target metX involved in methionine biosynthesis is found to be essential in all strains [59]. It is a proteinogenic as well as a component of S-adenosyl methionine which acts as a main methyl group carrier in cell. In most of the organisms including bacteria, it plays an important role in initiation of translation. Saint-Macary *et al*., 2015 reported that biosynthesis of methionine is essential for infection of *M*. *oryzae* [60]. Target cobA of cobalamin biosynthesis is present in chromosome I and also plays an essential role in the synthesis of vitamin-B12. Previous experimental evidence showed that vitamin B12 lacking growth medium was unable to grow the *L*. *interrogans* [50]. Thiamine monophosphate kinase (thiL) is an enzyme that catalyzes the thiamine monophosphate and converts it into thiamine pyrophosphate which is an essential cofactor in all living organism including *Leptospira* spp. Bian *et al*., 2011 discovered that to regulate the ABC transporters, riboswitches restricted to interact first with thiamine pyrophosphates due to which pathogen failed to grow in insufficient environment of thiamine pyrophosphate [52].

Thus, the proposed hub genes through the computational approach herein, support to find out broad spectrum drug targets that would be effective against pathogenic strain of *Leptospira* species. Although, the significance level of these putative targets need to be validated through experimental approach to ensure them as real one and can be the best against pathogenic strains *of Leptospira*.

## Conclusion

In the post genomic era, drug designing and discovery method is changing earlier established viewpoints. It routinely reorganizes the drug discovery method by incorporating vast data encrypted in our genome. We have performed subtractive genomic and comparative genomics analyses with network analysis of 16 pathogenic strains of *Leptospira* and identified 8 common drug targets that can be potential targets for drug designing and vaccine development. Moreover, many of the recognized drug targets have been observed to play a key role in the essential metabolic pathways, lipid biosynthesis, flagellar motor protein system and Bacterial chemotaxis. Among these targets, cobA and thiL also found as potent drug targets during genome scale metabolic reconstruction and choke point analysis. An efficient way to develop drugs against these targets would be substantially positive to diminish the threats of serious leptospirosis. However, these targets should be corroborated by further laboratory research for their role in inhibiting the growth and affecting the virulence of pathogens. Genome scale metabolic model of *Leptospira* would be useful in future for the basis of in silico gene knockout studies. Determination of qualitative tertiary structure and acknowledgment of functionally critical residues of these putative drug targets would be more effective in future for identification of novel leads and its optimization through *in silico* approaches like protein-ligand docking, free energy calculation and molecular dynamic simulations to design new anti-leptospiral drug against leptospirosis.

## Supporting information

S1 SBML Genome scale metabolic network reconstruction of *L*.*interrogans* serovar Copenhageni str. Fiocruz L1-130. (RAR)

S2 SBML Genome scale metabolic network reconstruction of *L*.*borgpetersenii* serovar Hardjo-bovis str. L550. (RAR)

S1 File Multiple sequence alignment of thiL. (DOCX)

S2 File Multiple sequence alignment of cobA. (DOCX)
