# Supplementary figures and images for: An in silico approach towards identification of novel drug targets in pathogenic species of Leptospira
Source: PLoS One. 2019 Aug 20;14(8):e0221446. doi: 10.1371/journal.pone.0221446 (PMC6701809; doi:10.1371/journal.pone.0221446)

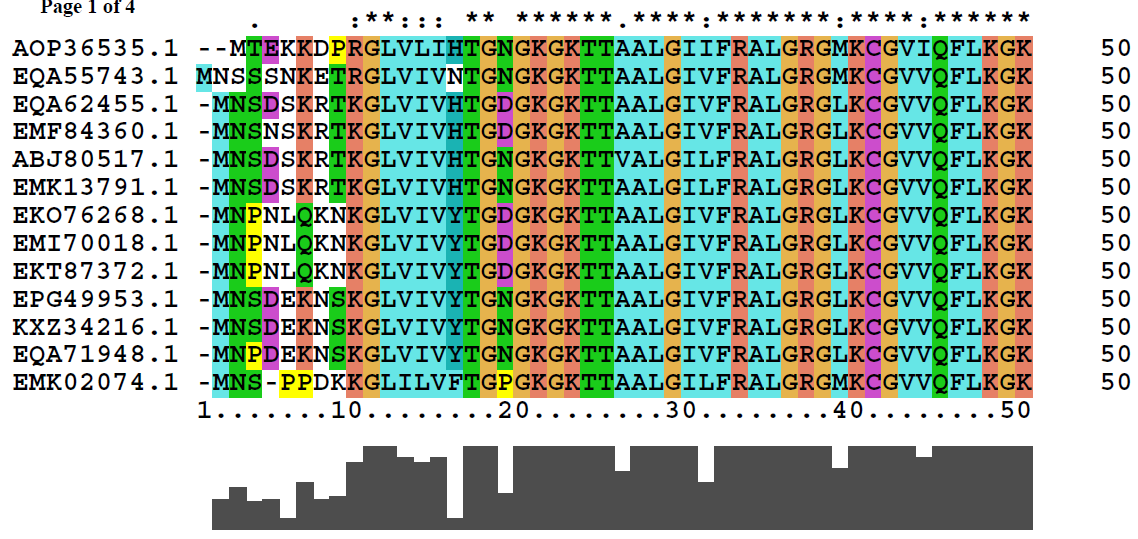


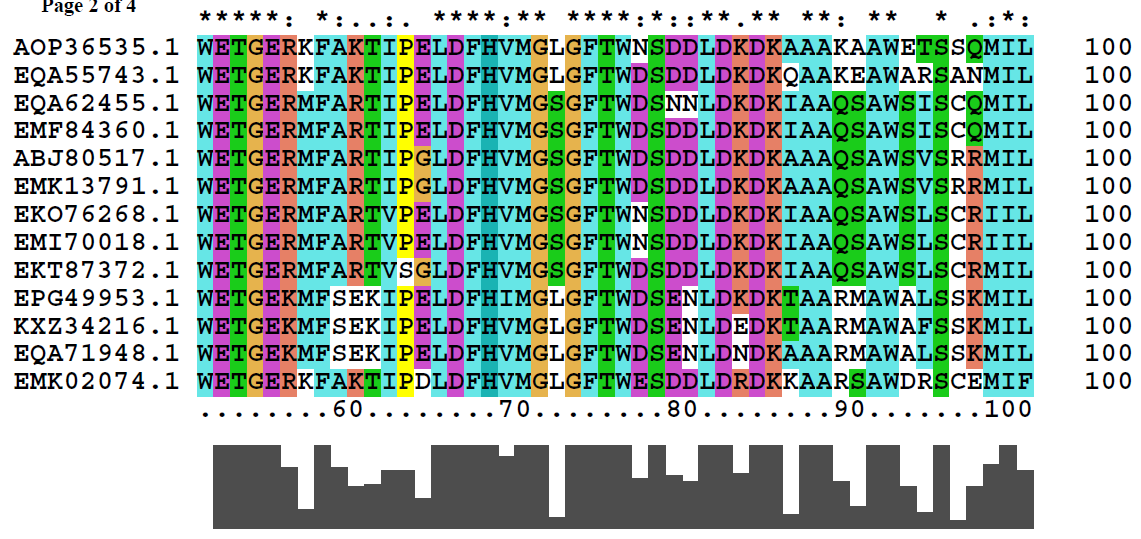


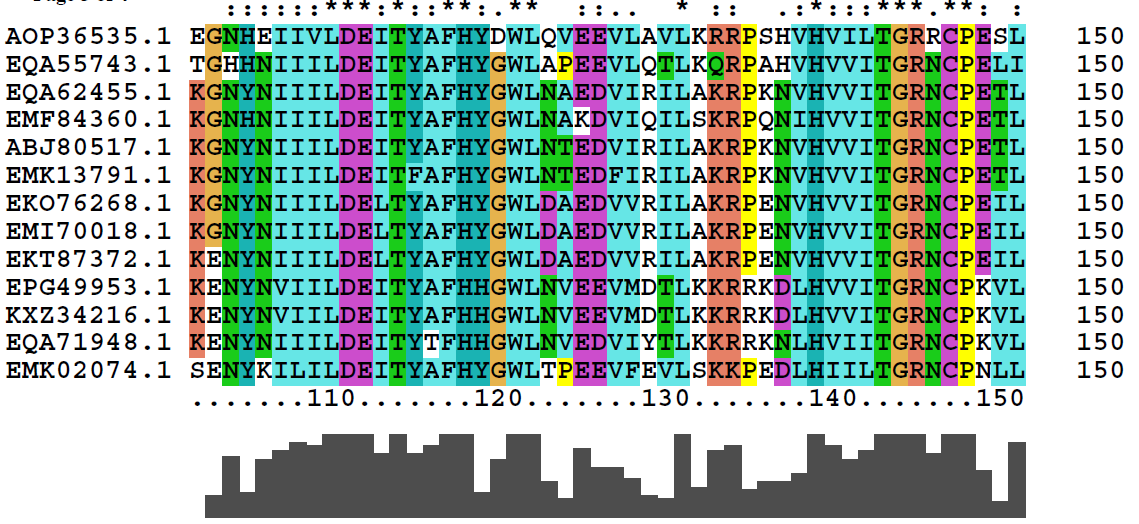


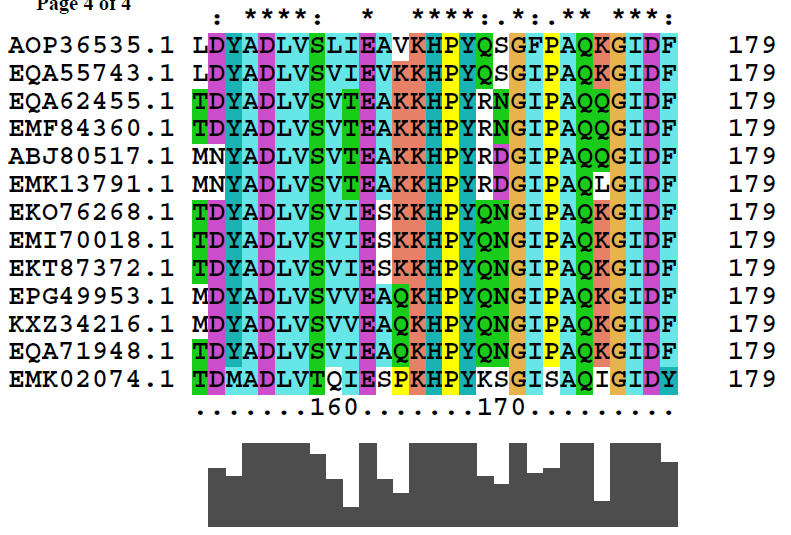

Supplement: S1 File — (DOCX) [file pone.0221446.s003.docx]

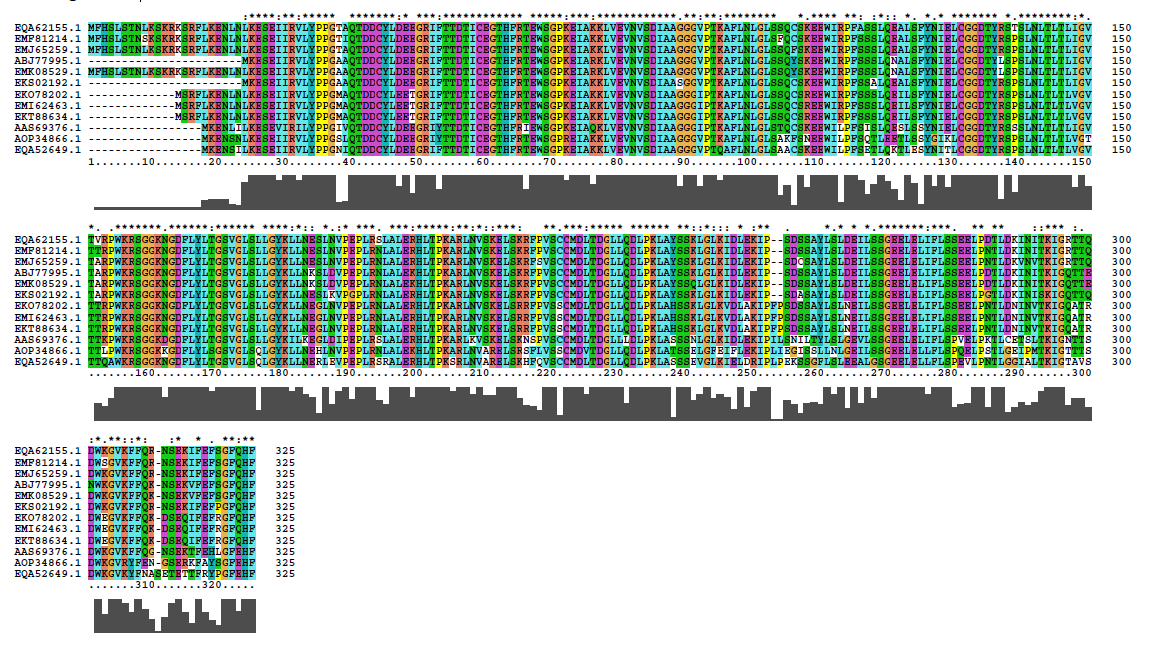

Supplement: S2 File — (DOCX) [file pone.0221446.s004.docx]
